# The Antiseptic Treatment of Summer Diarrhœa

**Published:** 1887-03

**Authors:** 


THE ANTISEPTIC TREATMENT OF SUMMER DIARRHŒA.—In an article on this subject (*N. Y. Med. Journal*, January 27, 1887), Dr. L. Emmett Holt sums up the following conclusions:

1. Summer diarrhœa is not to be regarded as a disease depending upon a single morbific agent.

2. The remote causes are many, and include heat, mode of feeding, surroundings, dentition, and many other factors.

3. The immediate cause is the putrefactive changes which take place in the stomach and bowels in food not digested, which changes are often begun outside the body.

4. These products may act as systemic poisons, or the particles may cause local irritation and inflammation of the intestine.

5. The diarrhœal discharges, *at the outset* at least, are to be looked upon as salutary.

6. The routine use of opium and astringents in these cases is not only useless, but, in the beginning particularly, they may do positive harm, since, by checking peristalsis, opium stops elimination and increases decomposition.

7. I do not deny or undervalue opium in many other forms of diarrhœa than the one under discussion.

8. Evacuants are to be considered an essential part of the antiseptic treatment.

9. Experience thus far leads me to regard naphthalin and the salts of salicylic acid as the most valuable antiseptics for the intestinal tract.

